# High Degree of Heterogeneity in Alzheimer's Disease Progression Patterns

**DOI:** 10.1371/journal.pcbi.1002251

**Published:** 2011-11-03

**Authors:** Natalia L. Komarova, Craig J. Thalhauser

**Affiliations:** Department of Mathematics, University of California Irvine, Irvine, California, United States of America; University of Colorado Denver School of Medicine, United States of America

## Abstract

There have been several reports on the varying rates of progression among Alzheimer's Disease (AD) patients; however, there has been no quantitative study of the amount of heterogeneity in AD. Obtaining a reliable quantitative measure of AD progression rates and their variances among the patients for each stage of AD is essential for evaluating results of any clinical study. The Global Deterioration Scale (GDS) and Functional Assessment Staging procedure (FAST) characterize seven stages in the course of AD from normal aging to severe dementia. Each GDS/FAST stage has a published mean duration, but the variance is unknown. We use statistical analysis to reconstruct GDS/FAST stage durations in a cohort of 648 AD patients with an average follow-up time of 4.78 years. Calculations for GDS/FAST stages 4–6 reveal that the standard deviations for stage durations are comparable with their mean values, indicating the presence of large variations in the AD progression among patients. Such amount of heterogeneity in the course of progression of AD is consistent with the existence of several sub-groups of AD patients, which differ by their patterns of decline.

## Author Summary

In recent decades, our understanding of Alzheimer's disease (AD) has increased; however, some basic questions still remain unresolved. One of them is: how homogeneous is AD? Is the course of progression more or less the same for most patients, or are there large variations? Our paper studies a large cohort of AD patients which comes from a 23-year-long study, and performs a statistical analysis of progression speed. We quantify the amount of spread in GDS/FAST stage durations (a staging system widely used by clinicians). We arrive at an astonishing conclusion that the mean length of AD stages is comparable with their standard deviation! This means that individual courses of AD progression may differ very much from each other, and from the textbook mean values. This has implications both for clinical trials (how do we assess if a new drug is effective, if the amount of natural spread is so large in untreated patients?), and for our understanding of this disease, which appears to be comprised of sub-diseases with different patterns of decline.

## Introduction

The temporal progression of Alzheimer's Disease (AD) shows a pattern of high variability, with patients transiting the stages of the disease having time-courses ranging from months to decades [1], [2]. While the biological correlates of this variability have been investigated by many groups [2]–[19], the underlying reasons for such variations remain largely uncertain. One of the challenges posed by a high variability of a temporal disease course is the difficulty in treatment efficiency assessments. For any current and future progression-delaying drug, it is important to be able to establish whether and by how much it delays the deterioration caused by AD. To this end, it is necessary to have a reliable quantification of the heterogeneity of the disease.

Global Deterioration Scale (GDS) was proposed in [20] and allows professionals and caregivers to chart the decline of people with AD. While a number of scales exist, GDS is one of the most widely used instruments to stage the course of AD. It measures cognitive, behavioral and functional impairment of patients. There are a total of 7 GDS stages (from stage 1 corresponding to no impairment to stage 7 corresponding to the most severe AD). In particular, stage 4 (mild AD) is characterized by patients requiring assistance in complex tasks such as handling finances, planning a dinner party etc. In GDS stage 5 (moderate AD) patients require assistance in choosing proper attire. In stage 6 (moderately severe AD) patients require assistance in dressing and bathing, and start experiencing urinary and fecal incontinence. GDS has been shown to correlate with both behavioral measures, and anatomic brain changes [20].

Functional Assessment Staging procedure (FAST) was proposed in Ref. [21], [22]. Based on GDS, this procedure describes a continuum of 16 successive stages and substages from normality to most severe dementia of the AD type. The FAST stages have been enumerated to be concordant with the GDS stages from which they were derived [23], although some differences between the two scales have also been demonstrated [24]. One of the advantages of GDS/FAST staging system is that it allows the assessment and staging of AD in its entire range from normal aging to very severe, end-stage, AD [25].

In the literature, the course of AD as characterized by GDS/FAST staging system has been described in quantitative terms. In particular, the stages are thought to follow in a sequential fashion and are characterized by certain stage durations [26]. For example, stage 4 is thought to last for 2 years, to be followed by stage 5 whose duration is 1.5 years, which in turn is followed by stage 6 (2.5 years).

While this quantification is a useful diagnostic tool, it reflects the average course of the disease and provides no information about possible heterogeneity of AD progression. At the same time, quantifying the variance of GDS/FAST stage durations is essential, as one needs to compare the delay gained by a treatment strategy with the amount of natural variation in stage durations, to be able to judge whether there is significance to any improvements observed. In this paper we investigate the heterogeneity of AD by studying the distribution of GDS/FAST stage durations of AD patients. We ask: how much variability is there in the course of AD, and how well do the average values for GDS/FAST stage durations reflect the disease course of individual patients?

## Results

**Figure 1:**
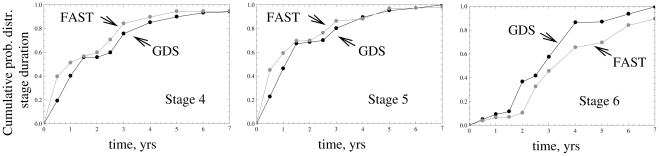
The calculated cumulative probability distribution functions for GDS/FAST stage durations.

**Figure 2:**
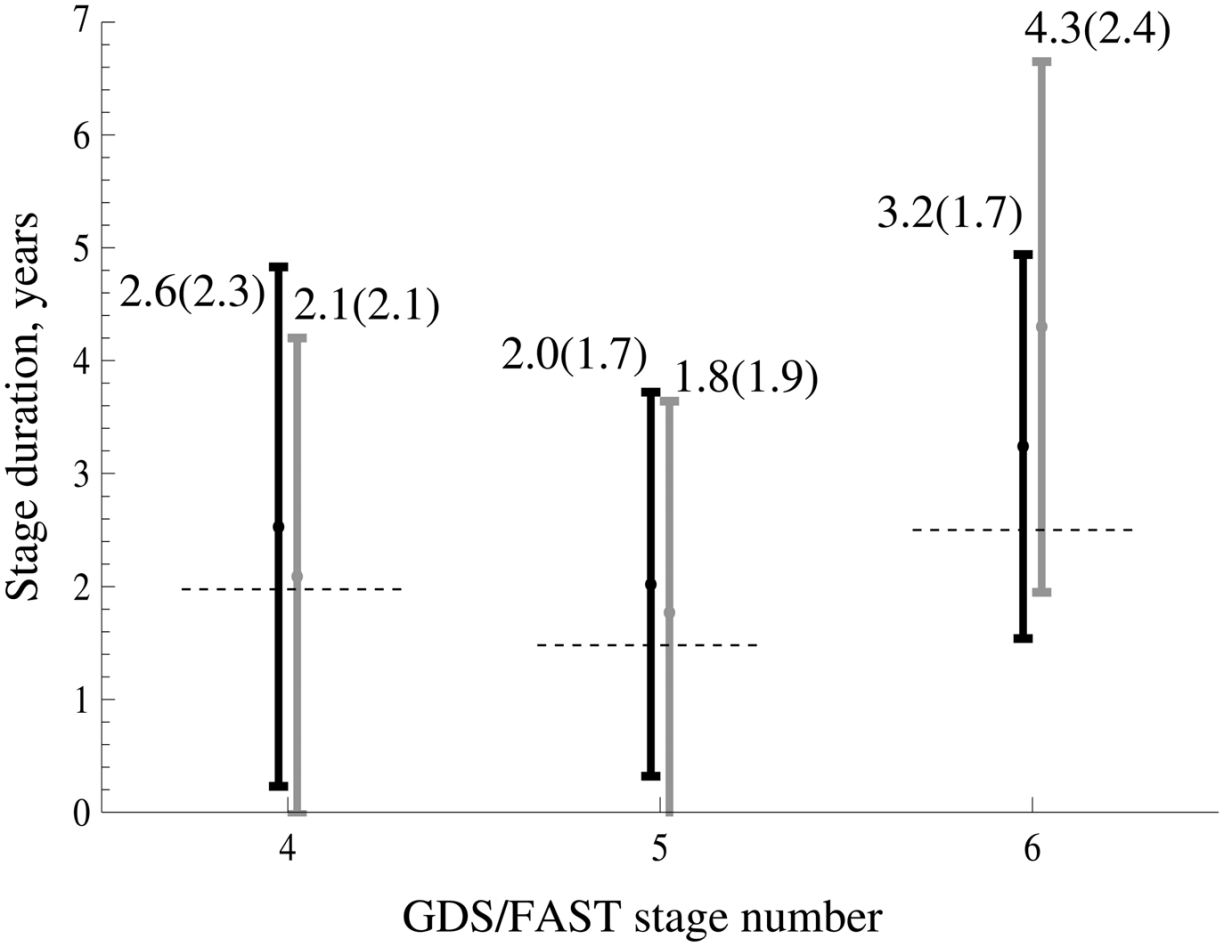
The mean values and standard deviations calculated for GDS/FAST stages 4–6. The black bars represent GDS stages, and the gray bars – FAST stages. The mean stage values reported in [27] are presented by dashed horizontal lines.

The estimates for the cumulative probability distributions of GDS/FAST stage durations are presented in figure 1. We can see that there is a slight difference between the GDS and FAST scale. This is further illustrated in figure 2 where we show the mean values of the GDS/FAST stage durations together with their standard deviations. In both figures, the values pertaining to the GDS system are plotted in black, and those for FAST staging are represented by gray lines. We can see that for stages 4 and 5, the FAST stage mean durations are slightly shorter than the GDS mean durations, and for stage 6, the FAST stage mean duration is longer than that calculated for the GDS system. We can also see that for stages 4,5 and 6, the estimated mean durations are somewhat longer than those given in [27] (the values from [27] for each GDS/FAST stage are shown by dashed horizontal lines). Despite this fact, we can see that, consistent with the literature, the GDS/FAST stage 5 is the shortest of the three stages, followed by stages 4, and 6.

A striking observation can be made by looking at the calculated values for the standard deviations of the stage durations. In figure 2, the standard deviation values are represented by vertical bars around the mean, and are also shown in brackets next to the calculated means. Both for GDS and FAST staging systems, the standard deviations are relatively large. For example, for the shorter stages 4 and 5, the standard deviations are of the order of the mean values for stage durations, and for the longer stage 6, the standard deviations exceed 50% of the mean stage duration values. Given such large standard deviations of stage length durations, it is remarkable that the calculated mean values of stages 4 and 5 are so close to the previously reported durations; and for stage 6, the calculated means are definitely within a standard deviation from the value in [27]. We further observe that the differences between the GDS and FAST measurements are also well within the standard deviation, so we cannot conclude that the two systems yield different mean values [25].

## Discussion

Analysis of a large longitudinal dataset has revealed a significant degree of variation in the lengths of GDS/FAST stages 4–6 of AD. In particular, the calculated standard deviations for GDS/FAST stage durations turned out to have values similar to their mean durations. This is an indication that the patterns of cognitive and functional decline vary significantly from patient to patient.

The suggestion that AD is a genuinely heterogeneous disease, has been proposed in the literature [28]. One paper [29] studies a 4-year longitudinal dataset, and identifies four different subgroups of AD patients which differ by the rate of their intellectual and functional decline as well as other symptoms. Ref. [30] states that AD shows heterogeneity in its clinical, anatomic, and physiologic characteristics, and identifies several patient subtypes with respect to different characteristics, including the time course of progression. In particular, inhomogeneity is observed with respect to the rates of ventricle enlargement, which are related to rates of cognitive decline. In Ref. [31], the presence of aphasia in AD patients is correlated with a more rapid course of the disease. This is done by performing extensive testing of the patients, as well as interviewing reliable informants, in the course of a 2.5 year-long follow-up. Ref. [2] follows patients for 3 year, and discovers an association between relatively severe frontal lobe involvement and a rapid clinical course of AD, measured by using the dementia rating scale and estimating the symptom duration time. A recent paper, Ref. [32], examines AD data from a 15-year longitudinal study, and provides important insights into the patterns of progression of AD. It identifies three groups of patients based on their initial (pre-progression) rate. This rate is estimated by using the (normalized) Mini Mental Status Exam (MMSE) score at base-line, divided by the symptoms' duration. It is found that the different groups remain separate in the course of the follow-ups, which is consistent with our previous finding [33]. Most relevant to our present study, it is found that the average rates of decline for the three groups are different for three types of measures: a cognitive measure (Alzheimer's disease Assessment Scale-Cognitive Subscale), a functional measure (Physical Self-Maintenance Scale), and a global measure (Clinical Dementia Rating Scale Sum of Boxes). Although no direct estimate of the variation has been presented, these results clearly show that AD progression rates are heterogeneous in many respects.

The patient data used here come from a longitudinal study conveyed between 1983 and 2006. It is theoretically possible that the large variation observed in the cohort of patients is a consequence of a change in lifestyle factors, which affected the course of AD progression. To explore this possibility, we have split the cohort of patients into two subgroups based on their dates of visit, and calculated the statistics of stage durations both for the “earlier” and the “later” parts of the cohort. We found that within the subgroups, the variances of the stage durations were as large as the ones reported here, and further, the mean values of stage durations were not significantly different.

Note, however, that the analysis performed here was not specifically designed to discern slight trends in the disease progression over the decades. We cannot perform such an analysis with the data at hand because of the data scarcity issues (using smaller sub-groups of patients necessarily jeopardizes the reliability of the statistics). More data would be needed to catch the trends related to changes in life-style and other generational effects. Here we could only conclude that in both early and late halves of the cohort, the variances were large, and stage durations were statistically not different.

Given a high variability of progression patterns, an important question is finding variables that correlate with progression rates. We have attempted to relate the rate of progression to demographic factors, and determine if it correlates with age at baseline,sex, education, or the age of onset of AD (which was back-calculated by using the information on the estimated stage durations). No significant correlations with these factors have been found, which is consistent with several previous papers [2], [13]–[19]. In the literature, several factors have been proposed to be predictive of the disease progression rate. The work of [34] highlights the heterogeneity of AD, and shows that clusters of CSF biomarker levels are related to patients' cognitive profiles. In particular, it finds that patients with extremely high CSF levels of tau and tau phosphorylated at threonine 181 demonstrate a distinct cognitive profile with more severe impairment of memory, mental speed, and executive functions; importantly, these differences cannot be explained by disease severity. Paper [35] finds that at the time of diagnosis, a combination of high CSF tau without proportionally elevated p-tau-181 is correlated with a faster rate of cognitive decline in AD patients. In paper [36], the variability of AD is explained in terms of specific types of EEG abnormalities. In paper [37], heterogeneity of AD is related to genetic variation in patients, such as that associated with cerebrospinal fluid phospho-tau levels. It is plausible that a combination of many different factors is responsible for a high variability of AD progression rates.

Our main finding is the large heterogeneity in the duration of GDS/FAST stages in AD, which is consistent with the reports cited above. Our methods however are very different. In this study we use a very extensive (23-year long) longitudinal dataset for AD patients, where there is a representation of patients at GDS/FAST stages 4–7 of AD. We calculate the amount of variance in patients explicitly, and demonstrate a large spread in values of GDS/FAST stage values for stages 4, 5, and 6. There are several applications of our results.

- Most immediately, having a standard deviation values (and not just the mean values) for GDS/FAST stage durations is important for those scientists and clinicians who use the GDS/FAST staging system.
- Such large values of variance in GDS/FAST stage durations caution against interpreting the GDS/FAST system as a prognostic tool: the course of decline of individual patients can be very different from the mean.
- Having the estimate on the GDS/FAST stage durations calculated in such an extensive longitudinal dataset shows the amount of heterogeneity in the course of progression of AD. This is consistent with the existence of several sub-groups of AD patients, which differ by their patterns of decline, see also [33].
- The knowledge of stage durations together with their natural variance is a necessary tool for the clinical trials. It allows to make quantitative judgments about new drugs’ efficiency.

To conclude, we analyzed a longitudinal dataset to extract the mean and the standard deviation for GDS/FAST stage durations for stages 4–6 of AD. Applying similar methodology to larger datasets with more frequent assessments will reveal more accurate results.

## Materials and Methods

**Figure 3:**
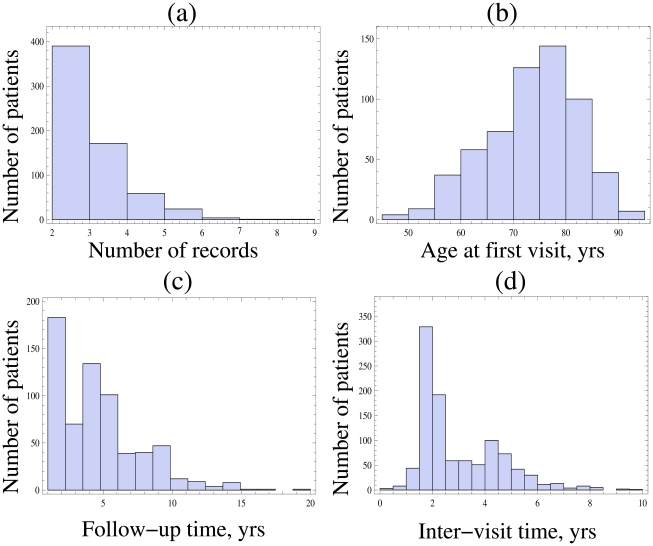
Some statistics of the dataset. (a) A histogram showing the number of records per patient. (b) A histogram showing patient inter-visit times.

In order to calculate the probability distribution of stage durations in AD, we used a longitudinal dataset of AD patients, which is an outcome of a longitudinal study performed between the years 1983 and 2006 [33]. The following information is contained in the dataset: the date of each patient's visit to the Medical Center, current GDS and FAST stage, and some demographic information on each patient (such as gender, age and years of education). The total number of AD patients in the dataset is 1321, of which 648 have repeated records (that is, they were seen more than once). The latter group is the one we considered in this study. The mean number of records per patient is 2.6±0.9; the histogram of the number of records for different patients is presented in figure 3(a). The patients' age at the first visit to the clinic is 73.1±8.7 years (see figure 3(b) for the age-distribution). 66% of the patients are female, and 34% male; the average length of education received by the patients is 13.1±3.4 years.

Extracting accurate estimates for the standard deviations for longitudinal datasets is complicated by the practical realities of how the data is collected. First of all, we only know the current stage at the times of assessments, but we have no information on when each stage actually starts and the next one begins (in other words, the data is left-and right- censored). Further complication comes from the fact that the patients' total observation time (time from first to last visit) was 4.78 ± 2.94 years, see the histogram of figure 3(c). This means that many patients in the cohort were not followed for the entire course of their disease. Table 1 shows a split of all the patients into transition classes, that is, it counts the number of patients first seen in stage *i*, and last seen in stage *j*. This quantifies exactly how many patients contribute to the calculations for different stages. It is obvious that the information coming from each individual patient is not nearly sufficient to reconstruct all the FAST/GDS stage durations. A method is required which would allow to combine data from different patients to reconstruct the stage duration distributions for the whole cohort (although the information coming from individual patients is incremental). Finally, another problem is illustrated in figure 3(d), where we present the inter-visit time distribution, which shows how long the patients waited before their next visit to the doctor. We can see that: (1) the distribution has a strong peak around 2 years, and then a weaker mode around 4 years, which tells us that the sampling times are strongly biased (the reason for this shape of the distribution is that the next appointment is usually recommended after two years); and (2) the average inter-visit time, which is 3.03±1.59, is comparable with the approximate average stage duration for FAST stages 4–6, which makes this dataset very “coarse” and not ideally suited for extracting stage time variations.

**Table 1:** The number of patients sorted by their FAST/GPS stage at their first and last visit.

*i* (down) and *j* (right)	3	4	5	6	7
3	3/0	8/1	8/1	7/0	3/0
4	0	50/73	58/90	108/88	75/81
5	0	0	33/34	98/95	60/90
6	0	0	0	58/23	67/61
7	0	0	0	0	12/11

The first column is the stage at the first visit, *i*, and the first row is the stage at the last visit to the clinic, *j*. The entries in the body of the table are numbers of patients whose first visit was at stage *i* and the last visit at stage *j*. The first entry in each cell corresponds to the FAST stages, and the second to the GDS stages.

Analysis of long, multistage disease processes has been addressed in literature in many different context [38]–[40]. Statistical approaches to estimating the mean stage durations from a set of AD patients medical records have centered on a linear regression approach [26], where the mean duration of FAST stages were determined, or the use of statistics such as the Kaplan-Meier estimate [32], [41] to determine the survival times of patients. Unfortunately, the linear regression method does not lend itself to calculating the variances of FAST stage durations (see Text S1). Here we used the methodology developed by [42]–[44] to approximate the probability distribution of stage durations.

We view the beginning and the end of each stage as censored events. For each stage *i*, for each patient, we identify the latest record when they were diagnosed with a stage prior to *i* (e.g. stage *i-1*), and then the earliest record where they were diagnosed with stage *i* or higher. These two time-points give us the interval of time where stage *i* began, *[X_L_,X_R_]*. Similarly, the latest record in stage *i* or lower, together with the earliest record at a stage higher than *i*, give the time-interval where stage *i* ended, *[Z_L_,Z_R_]*. Some of the right bounds are set to infinity for the lack of appropriate records. We further make an assumption on the patients' first visit, see Text S1 and also [33]: for patients who come to the doctor's office for the first time, we assume that the date of the visit effectively coincides with the onset of the current stage.

We used the iterative approach developed in [43] to approximate the probability distribution function of stage durations for stages 4, 5 and 6. We did not perform the analysis for stage 3 because the number of records for GDS/FAST stages 3 and lower was very small in the database. For stage 7, we were not able to extract meaningful information on the stage duration because of the absence of data on patients' death. The obtained solutions were further checked against a non-parametric numerical estimate of the cumulative distribution function obtained by a straightforward counting method. The two methods are mathematically different, but they revealed very similar results. Further details of the methodology are given in Text S1.

## Supporting Information

Text S1 Supporting information.
(PDF)
