# Supplementary material for: High Degree of Heterogeneity in Alzheimer's Disease Progression Patterns
Source: PLoS Comput Biol. 2011 Nov 3;7(11):e1002251. doi: 10.1371/journal.pcbi.1002251 (PMC3207941; doi:10.1371/journal.pcbi.1002251)
Supplement: Text S1 — Supporting information. (PDF) [file pcbi.1002251.s001.pdf]

High degree of heterogeneity in Alzheimer's disease progression patterns

Natalia L. Komarova and Craig J. Thalhauser

Text S1

1 Analysis of doubly-censored data

We implemented the method of [1] to extract the probability distribution of GDS/FAST stage durations in AD. From the longitudinal dataset in hand we obtained the bounds for the beginning (X) and the end (Z) times of stages 4-6, $X_L \leq X \leq X_R$ and $Z_L \leq Z \leq Z_R$. We performed the calculations for each stage. For a given stage, we disregarded all the patients whose last visit happened before the given stage, or those whose first visit happened after the end of the given stage. In some cases, the value Z_R was set to ∞ for the lack of appropriate data (right censored).

If a patient made his/her first visit at stage i, we assumed that the corresponding stage began sometime within the last $\tilde{t}$ years before the first visit (see [7]). This assumption was motivated by the fact that a transition to a new stage brings about new, noticeable symptoms which can spur a visit to the doctor. Including this assumption in the regression analysis in [7] yielded the mean stage durations remarkably close to those previously reported in the literature [5]. Unfortunately, this method cannot be extended to variance calculations (see below). For this reason, here we used a different approach to calculate the stage duration variance, but we kept the assumption that the first patients visit was soon after the beginning of the current stage.

In the figures of the main text, we present the results with the parameter $\tilde{t} = 0.3$ years. For stages 5 and 6, the calculated mean and variance values for stage durations did not vary significantly with the value of $\tilde{t}$. Table 1 shows the mean values and standard deviations of stage durations calculated by using $\tilde{t} = 0.3$ and $\tilde{t} = 0.6$. We can see that the results for stages 5 and 6 did not change by more than 0.1 year. However, results for stage 4 seem to depend more on the value of $\tilde{t}$. This is not surprising because there were very few records in the database from patients diagnosed with stage 3. Therefore, most information about the beginning of stage 4 is obtained from the records of patients whose first visit occurred in stage 4, and increasing the value of $\tilde{t}$ by a month would increase the calculated mean by up to a month. Note however that the fact that the values of the standard deviations of stage durations remain relatively large for different values of $\tilde{t}$, thus making this result independent on the choice of $\tilde{t}$.

The method of [1] requires splitting the time-axis into discrete intervals, both for the absolute value of stage beginning, $x_1, \ldots, x_r$, and for the stage duration, $t_1, \ldots, t_s$. We have arranged the time-points in such a way that each interval $[X_L, X_R]$ contained at least one value x_i, and each interval $[Z_L, Z_R]$ contained at least one value of $x_i + t_j$. We have checked that the results of the calculations do

$\tilde{t}$, yrs	GDS 4	FAST 4	GDS 5	FAST 5	GDS 6	FAST 6
0.3	2.57 ± 2.3	2.09 ± 2.11	2.03 ± 1.70	1.77 ± 1.87	3.24 ± 1.65	4.30 ± 2.35
0.6	2.78 ± 2.12	2.38 ± 1.89	2.09 ± 1.63	1.87 ± 1.84	3.27 ± 1.68	4.34 ± 2.30
Counting	2.48 ± 3.11	2.14 ± 2.76	1.89 ± 1.54	2.37 ± 2.29	3.42 ± 1.93	4.51 ± 2.55

Table 1: Mean values and standard deviations for stage durations (yrs) calculated by the method of [1] with different values of $\tilde{t}$. Also presented are the values calculated by the counting method, corresponding to $\tilde{t} = 0.3$ years and $n_{pat} = 5$.

not depend significantly on the choice of time-points (uniform and non-uniform grids), or their number.

Calculations can formally be performed for stage 7, but the iterations converge to a solution where all the probability values are zero except for the largest time-point. This is expected since we have no upper bound on the time when stage 7 finishes. For this reason we were not able to obtain any results for GDS/FAST stage 7 duration.

2 A counting method as an alternative estimate of the cumulative distribution function

In order to double-check the results obtained by the method of [1], we designed an alternative method to estimate the cumulative probability distribution function. This non-parametric method is based on a simple event-counting algorithm.

We consider all the patients whose first visit corresponds to stage i.[1] For a given stage, say, stage i, we calculate the numerical cumulative probability distribution of the stage duration, $P_i(t)$. For these patients, we assume that their first visit is at $t = 0$ (the onset of stage i is therefore on average at time $t = -\tilde{t}/2$). Consider all the patients who visit the doctor's office in some relatively short interval $[t_j, t_{j+1}]$, all of whom were at stage i at time 0. The $\Delta t_j = t_{j+1} - t_j$ is chosen to ensure that each patient within the grouping has exactly one visit in the interval. Then we can compute N_t^j to be the number of patients who, upon visiting the doctor between times t_j and $t_j + \Delta t_j$, transited to the next stage of the disease. Thus, for all these patients, the duration of stage i was less that t_j. Likewise, we can define N_s^j as the number of patients seen at the clinic in the time interval $[t_j, t_j + \Delta t_j]$ who remained in stage i; for all of these patients, the duration of stage i is greater than $t_j + \Delta t_j$. For all the patients who have an office visit in the interval $[t_j, t_j + \Delta t_j]$ we define $\bar{t}_j$

[1] Because of this restriction, we are not using all the data available. Despite this fact, we still have a relatively large number of records to perform the calculations (namely, 334, 219 and 84 records for stages 4, 5, and 6 respectively).

to be the mean time of these office visits, and compute $P_i(t_j) = \frac{N_s^j}{N_s^j + N_t^j}$. The ordered pairs $(\bar{t}_j, P_i(t_j))$ for $j = 1...M$ are thus a numerical approximation to the cumulative distribution function.

We further assume that the underlying probability distribution has finite support; that is, there exist some t_{min} for which $t \le t_{min} \Rightarrow P(t) = 0$ and t_{max} for which $t \ge t_{max} \Rightarrow P(t) = 1$. We specify t_{min} and t_{max} as follows: set $t_{min} = \text{floor}(t_1)$ and $t_{max} = \text{ceiling}(t_M)$. The choice of interval lengths Δt_j is somewhat arbitrary, and defines the discretization grid for the numerical approximation of the cumulative probability function. We chose this grid to be non-uniform because the distribution of intervisit durations of the patients in the dataset was highly non-uniform (see figure 3(d) of the main text). We chose the time-intervals such that the number of patients, n_{pat}, in each of the intervals was the same. This way we avoided having a time-interval with too few, or zero, visits. We tried different values of n_{pat} from 5 to 25 and found that the results for the mean and standard deviation values only weakly depend on the choice of n_{pat}.

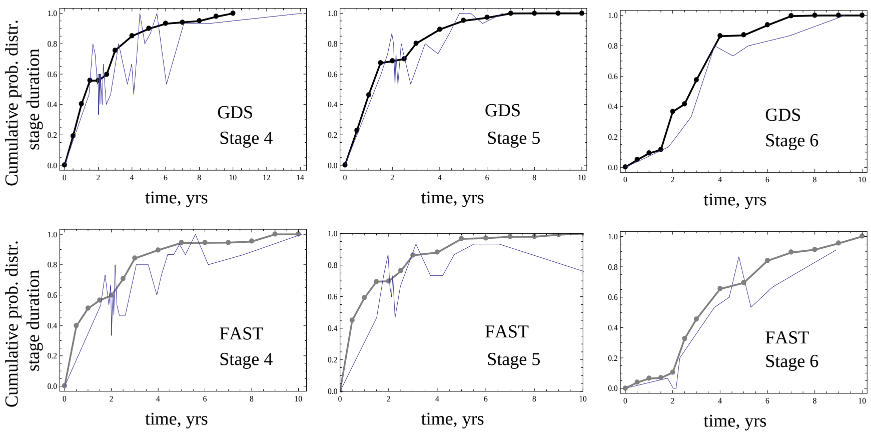

Figure 1: The comparison of cumulative probability distributions for stage durations obtained by two methods. The method of [1] is shown by thick lines with circles: black for GDS and gray for FAST stages. The counting method described here is presented by thin lines; we used $n_{pat} = 15$ and $\tilde{t} = 0.3$.

We compared the cumulative probability distribution function obtained by the method of [1] with that of the counting method described here. The results are presented in figure 1. We can see that the iterative method described in the previous section converges to solutions close to those obtained by the counting method. This means that the iterative method did not converge to a spurious solution. The last row of table 1 presents the mean values and standard deviations of stage durations calculated by the counting method. Again, they are similar to those obtained by the method of [1]. In particular, the result that the standard deviation values are relatively large, is confirmed by these calculations.

The advantage of the simple counting method is that it naturally gives a way to estimate the cumulative probability distribution of stage durations, in a very simple, non-iterative way. The disadvantage is that to get accurate results, we need to have a relatively large number of patients; with the amount of data available to us, the obtained solution is rather noisy, see figure 1.

3 The linear regression method

Finally, we briefly review the regression method used in [5, 7], and explain why it was not used in the present study.

The first step in this method is to classify each patient based on their medical record, that is, at which stage they came to the doctor at their first and subsequent visit (for a disease with n stages, there are $\frac{n(n+1)}{2}$ possible transition classes). For a patient in transition class $i \to j$, with $i \leq j$, the time elapsed between two doctor's visits is given by $t_{ij} = \sum_{s=i}^{j} x_s T_s$, where T_s is the duration of stage s for the given patient, and the completion coefficients, x_s, satisfy the following: $x_s = 0$ if a patient did not enter stage s; $0 < x_s < 1$, if a patient did not fully complete stage s; and $x_s = 1$, if a patient fully transited stage s. The values T_s and x_s are unknown, and the values t_{ij}, are determined from the patient data set.

We assume that T_s is a random variable with an unknown distribution, and that the completion coefficients for partial stage completion are distributed as $U(0,1)$, independent of T_s. Define a new random variable $Z_i = x_i T_i$, whenever a patient starts but does not fully complete stage i in the time-interval between the two visits. Since each of the two random variables in Z_i is independent of one another, we have

$$\begin{aligned} E[Z_i] &= \frac{1}{2} E[T_i], \\ Var[Z_i] &= \frac{Var[T_i]}{3} + \frac{E^2[T_i]}{12}. \end{aligned}$$

Therefore, for a patient set entering at stage i and exiting at stage j, with total time-interval $t_{i,j}$, the total mean and variance can be calculated as:

$$E[t_{i,j}] = \frac{E[T_i]}{2} + \sum_{s=i+1}^{j-1} E[T_s] + \frac{E[T_j]}{2}, \tag{1}$$

$$Var[t_{i,j}] = \frac{Var[T_i]}{3} + \frac{E^2[T_i]}{12} + \sum_{s=i+1}^{j-1} Var[T_s x] + \frac{Var[T_j]}{3} + \frac{E^2[T_j]}{12}. \tag{2}$$

In the case of an $i \to i$ transition, only the first term in each equation is used.

Under the assumption that the patients from transition classes $i \to j$ with $j > i$ tend to make their initial visit at the beginning of a stage, rather than

in the middle or end of that stage, the completion coefficient for classes $i \to j$ with $j > i$ is given by 1. This results in the following system:

$$E[t_{i,j}] = E[T_i] + \sum_{s=i+1}^{j-1} E[T_s] + E[T_j]/2, \quad j > i, \tag{3}$$

$$E[t_{i,i}] = E[T_i]/2. \tag{4}$$

There are two drawbacks of the regression method that precluded us from using it in the present paper. One inherent problem is the assumption of independence of the completion coefficients and the patients' stage durations. Extensive testing using artificial data sets showed that despite this fact, the method gives reasonable predictions for the mean stage durations. However, it cannot be extended to calculating the variance, where the inherent interdependence of the completion coefficients and stage durations causes the current method to be very inaccurate. In a validation test of the regression method using a data set in which each stage of a 4-stage disease were i.i.d. with mean 3 and variance 1/3, with 1000 total patients in the set, the method produced a 95% confidence interval calculating the mean to 7% accuracy for each stage, yet the 95% confidence interval for calculating the variance was at best 20% for each stage.

Another assumption that the regression method implicitly makes is the independence of different stage durations. In the light of our previous analysis [7], as well as many other reports (see e.g. [2]), it appears that there are certain subgroups among AD patients that differ by their progression patterns. In particular, it has been suggested that slow progressors remain slow, while rapid progressors remain rapid. This means that the durations of different GDS/FAST stages are not independent. Therefore, the regression method should not be used without first separating the patients in different classes, as was done in [7].

4 Studying long-term trends

The patient data used in this study come from a longitudinal study conveyed between 1983 and 2006. An important question is the constancy of the diagnostic and clinical process in the course of 23 years. Both GDS [3] and FAST [4] staging procedures have been fully developed by 1986 (the start of the dataset). The reliability of the FAST staging has been studied in [6]. Different raters were asked to independently determine the AD stages of a number of patients, and such variables as "rater agreement" and "rater consistency" were evaluated and found to be "excellent". It was concluded that "FAST is a reliable and valid assessment technique for evaluating functional deterioration in AD patients throughout the entire course of the illness".

Next we examine the patients in terms of their progression stages and ask if there are any significant differences between patients that came early in the study and late in the study. We split all the patients in the dataset into two parts: the patients whose first visit occurred before 1994 (early cohort), and

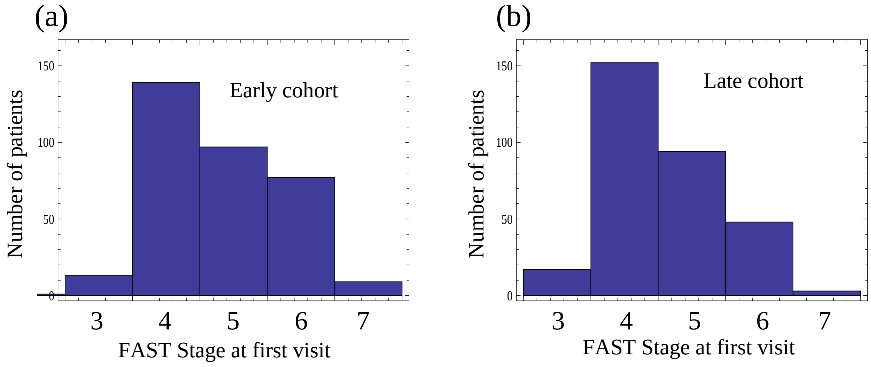

Figure 2: A comparison between early (a) and late (b) cohorts of the patients. The histograms represent the FAST stages at the first visit to the clinic.

those whose first visit occurred after 1994 (late cohort). There were 335 and 313 patients in the two cohorts respectively. In figure 2(a,b) we present histograms of progression stages at the fist visit, recorded for the patients in the early and the late cohorts. We can see that the distribution is similar. When we examine the histograms of all the stages recorded for the patients in the two subgroups (not shown), we can see that there is a difference. Namely, there are proportionally fewer patients seen in late stages of AD in the late cohort, compared to those in the early cohort. This is the consequence of the fact that in the late cohort, some of the patients did not progress to later stages before the end of the study (see also (a) at the end of this section). Thus the early and the late cohorts of the patients do not demonstrate any significant differences in terms of stage distributions.

Further, it is theoretically possible that in the course of 23 years the progression rates have changed. A significant change in progression rate could explain the large variance we are measuring in the whole cohort, which combines the data from all the patients. To eliminate this possibility, we have performed the following analysis. We used the two cohorts described above, and performed the analysis of stage durations separately for each subgroup. Note that for the patients in the early cohort, some of their subsequent visits may have happened after 1994, but the majority of the visits falls in the first half. We have calculated the mean stage durations together with their standard deviations for stages 4-6 for the two groups. It turned out that

(i) the standard deviations of stage durations in each group were similar to each other, and similar to the ones calculated for the whole cohort, and

(ii) the mean values of the stage durations in the two groups were not significantly different.

We also performed the following additional tests. (a) In the first patient group described above, we ignored all the visits that happened after 1994, and calcu-

lated the statistics of this group, compared to those of the second group (the patients whose first visit happened after 1994). (b) We also took random samples of all the patients regardless of the timing of their first visits, and computed the statistics for such groups. In both cases, conclusions (i) and (ii) held true.

References

[1] De Gruttola, V. and Lagakos, S. (1989) Analysis of Doubly-Censored Survival Data, with Application to AIDS, Biometrics, 45, 1-11.

[2] Doody, R.S., Pavlik, V., Massman,P., Rountree, S., Darby, E., Chan,W. (2010) Predicting progression of Alzheimer disease, Alzheimer Research & Therapy, 2, 1–9.

[3] Reisberg B, Ferris SH, de Leon MJ, Crook T (1982) The Global Deterioration Scale for assessment of primary degenerative dementia. Am J Psychiatry 139: 1136-1139.

[4] Reisberg B (1986) Dementia: a systematic approach to identifying reversible causes. Geriatrics 41: 30-46.

[5] Reisberg, B., Ferris, S. H., Franssen, E. H., Shulman, E., Monteiro, I., Sclan, S. G., Steinberg, G., Kluger, A., Torossian, C., de Leon, M. J. and Laska, E. (1996) Mortality and temporal course of probable Alzheimer's disease: a 5-year prospective study. Int Psychogeriatr 8, 291-311.

[6] Sclan SG, Reisberg B (1992) Functional assessment staging (FAST) in Alzheimer's disease: reliability, validity, and ordinality. Int Psychogeriatr 4 Suppl 1: 55-69.

[7] Thalhauser, C.J. and Komarova, N.L. (2011) Alzheimer's disease: rapid and slow progression. Jour. Roy. Soc. Interface. Epub Jun 6.
